# Vaginal Hysterectomy—Report of Three Cases

**Published:** 1888-09

**Authors:** J. H. Etheridge

**Affiliations:** Chicago, Ill.; Gynecologist to the Presbyterian Hospital and to the Central Free Dispensary


### VAGINAL HYSTERECTOMY—REPORT OF THREE CASES.*

BY J. H. ETHERIDGE, A. M., M. D. (RUSH), GYNECOLOGIST TO THE PRESBYTERIAN HOSPITAL AND TO THE CENTRAL FREE DISPENSARY.

The three cases reported herein were operated on at the Presbyterian Hospital. I can never convey an adequate idea of the relief to the operator offered by the method of hemostasis by forcipressure on the broad ligaments over that of ligatures. I think no one can fully appreciate the untold superiority of the former method over the latter till he has had experience in the performance of that operation under both methods.

CASE 1.—Mrs. S., æt. 47, mother of nine children, always well, presented herself February 1, 1887, with epithelioma of the cervix uteri. It did not involve the vault of the vagina. The broad ligaments did not seem to be thickened. Mobility of the uterus was complete. After preparatory treatment with a daily laxative and diuretic for a week, the operation was performed on February 8, 1887.

The cervix was easily drawn down to the vulvar orifice and, with scissors, its vaginal attachment was divided. Strong adhesions to the bladder and rectum were found, and in consequence thereof, the rectum was opened in one place and the bladder in two places in the process of freeing the uterus from these two organs. After the two broad ligaments were sufficiently isolated and the fundus was turned backwards and brought down, the left broad ligament was first penetrated and divided into two sections with heavy ligatures, and tied as securely as hands could tie them. It was then severed as closely to the corpus uteri as possible, and the whole organ came out of

*Read before the Chicago Medical Society.

the vagina. Treating the right broad ligament similarly was a much easier matter, because the uterus was down and out of the way. This attachment was at once severed, and the whole organ was then freed from the patient. The ovaries were then removed. The rent in the bowel was closed by continuous suture without difficulty. The longer rent in the bladder was then closed by continuous suture, but it was done at a great disadvantage from its peculiar position, back of the symphysis and looking directly backwards. To draw down the bladder and to so evert the edges of the rent as to apply the stitches was a delicate and difficult task. The smaller rent, undiscovered at that time, was not closed. Just as this sewing up was completed, there was observed, welling up into the shapeless excavation left after the removal of the uterus, great quantities of arterial blood. Which broad ligament it came from it was impossible to decide. After a long time the bleeding vessel, which was in the right broad ligament, was secured, but not till after a ligature was pushed off of the left broad ligament. All vessels were eventually secured, but not till a great quantity of blood had been lost. The top of the vagina was closed from before backwards with continuous suture, the ligatures were brought down, iodoform gauze stuffed into the vagina and the patient put to bed.

Reaction followed reluctlantly. She died from peritonitis and exhaustion in 45 hours, having passed 8 ounces and 1 drachm of urine in the meantime. The autopsy revealed a small rent in the bladder, which was concluded to be the cause of the peritonitis.

CASE 2.—Mrs. C., æt. 36, laundress, tall, spare, nervous, sanguine temperament, presented herself February 10, 1887, with a small epithelioma in the cervix uteri. The upper portion of the vaginal cervix was not invaded. The choice between amputation of the cervix and hysterectomy was left to the patient after a full explanation of the dangers and results of the two procedures. She decided to have the latter operation, which was performed on February 25, 1887.

The uterus was easily drawn down to the vulvar orifice and freed from its vaginal attachment with the scissors. The bladder was uncommonly closely attached to the uterus, and before its complete separation was accomplished it was opened. The opening into the Douglas cul-de-sac was easily effected, and the fundus rocked backward through the sacral hollow, down and out through the vulva. The broad ligaments were secured with silk ligatures, and the uterus removed after its separation from them. The ovaries were separately removed immediately afterwards. In closing the vesical rent the left broad ligament shed its ligatures and bled profusely. Hemorrhage was soon checked. The vagina was closed from before backwards, the ligatures were brought down into the vagina, and the latter organ was filled with iodoform gauze.

The patient rallied well. The temperature rose to 100° on the second and third days. Thereafter nothing worthy of special mention occurred. On the tenth day an elastic ligature attached to the patient's left thigh was tied to those protruding from the vagina, and in five days they began to come away, and in 48 hours the last one was removed. In 36 days she left the hospital.

CASE 3.—April 13, 1887. Mrs C., æt. 48, widow; last confinement 28 years ago; she is still menstruating regularly every three weeks, flowing one week. Six months ago she began to have leucorrhœa and to lose occasional small amounts of blood. She has excellent general health, digests well, and is a good excreter from the bowels, kidneys and skin. She is well nourished and presents a promising outcome for any surgical ordeal. The only thing that one could wish different in her general aspect is a too rapidly acting heart. It beats over 90 times per minute, and the arterial impulse is persistent. She has often seen lateritious deposits in her renal secretion.

Examination reveals an epitheliomatous degeneration of the cervix, with about ¼ inch of uninvaded tissue of the cervix between the cancer and the vaginal vault. The uterus was about four inches deep and it bled freely upon withdrawing the sound. The fundus was large and was easily felt through the abdominal wall. The uterus was freely movable, indicating the non-application of the lymphatics in the broad ligament. The absence of invasion of the vaginal wall and of the circumuterine tissues led to the recommending an operation for the removal of the entire uterus.

From April 19 till May 5, the date of the operation, she took cascara daily and digitalis and acetate of potash. The condition of the excretions seemed as nearly perfect as possible preparatory to an operation. The patient slept in the hospital the night before the operation, and took the customary antiseptic bath and had administered several vaginal bichloride douches.

*Operation.*—The cervix was drawn into the vulva with two large lock, vulsella forceps, while the vaginal attachment to the cervix was divided with the scissors. Gradually and patiently the circumcervical tissues and the attachments of the bladder and rectum were crowded away with the finger-nail till the Douglas cul-de-sac could be opened. Then it was found quite impossible to reach the top of the fundus with the fingers. The cul-de-sac of peritoneum between the bladder and the uterus was then opened, with hope of being able to retroflex the uterus by means of the fingers placed before and behind the uterus. This manœuvre was found likewise to be an impossibility. After repeated vain attempts to reach the top of the fundus with the fingers, that method was abandoned. Trial of very deep supra-pubic pressure to thrust the fundus back toward the sacral hollow, and at the same time of grasping and pulling down the fundus with a small vulsellum forceps thrust through the Douglas cul-de-sac, at last succeeded, after three or four tearings out of the forceps, in getting the top of uterus out into the world.

Snap forceps were then placed on the broad ligaments and the latter divided. The subsequent dressing consisted in tucking a thin layer of iodoform gauze into the vagina, care being taken to avoid separating the top of the vaginal walls. The danger of this separation must be patent to any observer. Ribollett attributes the death of one of his patients to crowding too much gauze into the upper vagina.

No stitches were used to close the upper end of the vagina. Its borders were permitted to collapse and to close in any position that they chanced to occupy. The fear that the bowels and bladder might seek an outlet through the vaginal tract is wholly groundless. One ligature was used for a vaginal artery. No attention was paid to it in the final dressing. The ovaries were both removed after the uterus was finally separated from its attachments.

The patient reacted well from the shock of the operation, which consumed seventy-five minutes. Her daily progress was so uniformly satisfactory that any detailed descriptive statement of it would be monotony itself. The pulse ranged from 90 to 120 beats per minute; it was 98 when she left the hospital. The temperature reached 100° one *morning* only, and on five evenings, from the third to the seventh day inclusive.

The amount of urine passed daily during the first fourteen days after the operation is indicated by ounces in the following figures: 19, 19½, 25, 23, 26, 24, 23½, 25, 30, 31½, 34, 40, 5, 26½.

The forceps, one pair on each ligamentum latum, were removed at the end of 48 hours. Although any one knows that 48 hours of obliteration of the lumen of an artery must necessarily destroy its potency, yet the writer was filled with misgivings when the forceps were very carefully unwrapped and as carefully removed as gentleness itself could supervise. The folds and creases of the vagina, as far up into the shapeless excavation as vision could peer, upon an exaggerated separation of the vulva with the fingers, were watched with an intensity of eagerness and anxiety, for their being inundated with the hot scarlet blood that so easily comes from the ovarian and uterine arteries, that can be appreciated only by him who has experienced those emotions. No bleeding followed the removal of pressure from the uterine ligaments. The vagina was not again filled with gauze. Iodoform was blown into this cavern as far as was possible daily.

It was impossible to state definitely the amount of drainage that escaped. Perhaps 3 tablespoonfuls daily for the first two days would cover it; afterwards the amount could not have been more than 1 tablespoonful on the third and fourth days each. After the fourth day none escaped to mention.

CASE 4 came to me several weeks ago for an incoercible hemorrhage. The patient, a woman 47 years of age, has borne eleven children, and has been having hemorrhage for eleven years.

Everything in the way of medicine has been tried; the uterus has been curetted upon two different occasions, and she came to me supposing the ovaries must be taken out. I explained to her that I had had two cases of removal of the ovaries for hemorrhage, but that the operation had not cured the hemorrhage. I also explained to her the danger of removal of the uterus, and she consented to the operation. The steps were simplicity itself, being a repetition of what I have given. The cervix was easily drawn down to the vulvar orifice and the cervical attachments freed at once as speedily as possible. An incision was made in the cul-de-sac of Douglas, and I was able with a knife to divide the tissues without any trouble. I kept as close to the uterus as I could, and did not open the bladder. When the two broad ligaments were all that supported the uterus, they were secured with long snap forceps and the broad ligament divided on the right and left sides. Snap forceps were put on the neighboring deep vessels; that was all the hemostatic means that was resorted to from beginning to end.

The patient was put to bed about 3 o'clock, and about 6 I was telephoned that she was having a severe hemorrhage. Concluding that it was one of the arterial branches from the vagina, I hoped that ergot would stop the hemorrhage. When I reached the hospital I found that after the use of the rectal injection of ergot the hemorrhage ceased. The case ran along very well for 48 hours; afterwards the temperature was 99° for two or three days and then it ran up to 101°. She was put upon the table and the wound exposed with a Sims speculum. Up in each angle or corner of the wound was a black gangrenous-looking mass about as big as my little finger, hanging down upon the vagina, which mass protruded from a little cavity there filled with the debris of blood, which had probably been produced by the hemorrhage on the day of the operation. It had decomposed and was smelling very badly. That mass was pulled out. In the other side I was able to pass the forceps up a distance of about 1½ inches. I got out all the shreds and put the patient to bed. The temperature at once went down to 98.2°, and since that time everything has progressed satisfactorily. This is the twenty-first day since the operation. I saw the patient this afternoon, and she calls for solid food and is to sit up to-morrow. The following are some points of interest concerning this operation:

*1. Indications for its Performance.*—(a) Ten years ago, and indeed until quite recently, the chief indication for the performance of vaginal hysterectomy was malignant disease. At present it is agreed by all operators that the earlier it is performed for cancer the greater are the chances for its non-recurrence. This dread malady always returns sooner or later after amputation of the uterine cervix, and of course proves fatal; whereas, when the whole organ is removed, the patient is given the only hope of a permanent recovery. Hysterectomy does not always prevent recurrence of the development of this neoplasm, yet it offers the best results. Martin reports 8 cases of hysterectomy from cancer without relapse, varying from two and one-half to five years. According to Sänger the average of survival after this operation is eleven months. Olshausen reports cases after operation of relapse, once after eighteen months, twice after two years, and twice after three years.

The most favorable conditions offered for hysterectomy are the non-involvement of the vagina, and complete mobility of the uterus, which shows the non-invasion of the *ligamenta lata*. In other words, the earlier in the disease the operation can be performed the better are the promises of a radical cure. Only too often does it occur that the disease has advanced too far before the gynecologist is consulted.

(b) *Procidentia uteri* is another condition for which this operation is performed. Anaplastic operations do not always restore the organ to its normal level. Artificial vaginal stenosis to the extent of the non-admission of the little fingei has failed ultimately to relieve the procidentia through gradual dilatation of the vaginal channel.

(c) Fibrous bodies of the uterus which offer the point of departure for serious irregularities have constituted a cause for vaginal hysterectomy. Of course reference is had to small tumors. Heydenreich reports four cases of operation with four successes. He[a] considers that at present it is impossible even to pronounce upon the relative merits of vaginal hysterectomy and of castration for small fibrous bodies in the uterus. Péan[b] recently reports a case of the same operation for multiple fibroids.

(d) The hystero-neuroses (inveterate dysmenorrhœa, neuralgia, convulsions, etc.), for which oöphorectomy is so often performed, Péan considers a justifiable cause for this surgical procedure. His reasoning is that these neuroses sustain an intimate relation to the uterus itself; consequently the uterus should be included along with the tubes and ovaries (Caldwell. Paris Letter in *Chicago Medical Journal and Examiner*, February 1887).

As an illustration of the *furor operations*, a recent article from the pen of a Cologne surgeon, Dr. Frank, published in April 3, 1887, number of the *Archiv für Gynäkologie*, enumerates the following cases of removal of the entire uterus: For endometritis, four cases; for retroflexion or retroversion with fixation, three cases; *pruritis uterinus*, once; and for neuralgia and retention of urine, one case. The members of the medical profession can scarcely read the account of these cases without being astounded at the amazing temerity of such proceedings.

The various steps in the operation consist in, 1, freeing the cervix from its attachments; 2, hemostasis; and 3, the subsequent dressing.

1. The cervix must be drawn down with forceps into the vulvar orifice, if possible, and the vaginal attachment severed with any cutting instrument, a bistoury, a blunt or a sharp-pointed scissors. Some operators prefer one instrument, others another. It is a trifling choice to make between them. The vulva should be held open laterally by retractors deftly held just within the ostium; if they are thrust into the vagina too far they prevent the forced descent of the uterus. If they are wide enough a perineal retractor is unnecessary. Just before making the initial cutting it is well to push up the cervix (which has been drawn down) to

---

[a] Albert Heydenreich. De l'hysterectomie Vaginale. Semaine Méd., Paris, 1886, vi. 69, 70.

[b] Gazette des Hôpitaux, October 12, 1886, pp. 950, 951.

its natural level and mark with the eye where the vagina is attached, and then draw down the organ and begin proceedings. This point is rather important, because no one can tell where the vaginal wall terminates and the cervical covering begins, and one is invariably inclined to begin the enucleation too far away from the cervix and thus to open the bladder. The encircling of the cervix can be made at once with tissues pushed away from the uterus in all directions till the broad ligaments are reached, when they will, of course, not be disturbed. The gravest necessity exists for keeping exceedingly close to the cervix anteriorly; otherwise the operator will find that he has opened the bladder almost before he has any idea that he is dangerously near it. By keeping as closely to the cervix as possible another important, nay vital, advantage will be gained, viz., the avoidance of wounding the ureter, which perforates the bladder just above the middle of the anterior vaginal wall. Wounding this duct complicates matters most wofully, in that it necessitates the extirpation of the kidney. The surest way of determiinng the dangerous proximity to the ureter is to discover the pulsation of its accompanying artery, which is a branch of the uterine artery, and is of considerable magnitude. Absolute safety from wounding this important channel is guaranteed to him only who keeps closely enough to the cervix in its denudation. Very soon the finger can be made to penetrate the peritoneal cavity, as will be indicated by its feeling the fundus covered with the smooth, glistening peritoneum. The freeing the posterior cervical wall should be prosecuted with the same care to remain close to the uterus and thus avoid opening the rectum. The finger easily penetrates the Douglas *cul-de-sac* and the body of the uterus can then be explored readily. Up to this point, when the peritoneal cavity is opened, the bleeding is considerable, though not at all alarming. It is best to proceed as rapidly as possible and not to attempt to check hemorrhage. Occasionally the peritoneum is tough and cannot be perforated by the finger; then a blunt-pointed pair of scissors, *closed*, can be thrust into this cavity, quickly opened and withdrawn, leaving an opening large enough to admit the finger.

After opening the posterior *cul-de-sac* some operators push a soft sponge into the peritoneal cavity, to remain there till the operation is terminated, for the purpose of preventing the entrance of noxious matters and of keeping the bowels up and away from possible injury. It also serves the purpose upon its withdrawal of drawing down the ragged edges of the peritoneum so that in the wound peritoneum lies opposed to peritoneum, a most desirable position to be secured.

At this point two proceedings lie open: one is to bring the fundus down through anterior cul-de-sac or through posterior cul-de-sac—*i. e.*, to acutely and completely anteflex the uterus—and the other is to let flexion entirely alone and to proceed at once with the treatment of the *ligamenta lata* with reference to preventing other vessels from bending and dividing them, and thus freeing the uterus wholly from the body. Another plan resorted to before removing the organ has been, after securing the broad ligaments, to bisect the uterus from fundus to os, and removing each half separately. It must be a very exceptional case demanding this proceeding. When the uterus is small, flexion is an easy matter. When large it is a very difficult matter, and when very large it is a feat impossible to accomplish.

C. Staude[a] recommends opening the Douglas cul-de-sac first and retroflexing the uterus completely before opening the vesico-uterine cul-de-sac, in order not to permit the cancerous cervix to enter the peritoneal cavity as the fundus is brought downwards. The ante-uterine peritoneal space thus shut off will effectually prevent the cervix entering it. However, with the cervix firmly held by the vulsellum forceps, it is impossible for it to ascend into the peritoneal cavity as the fundus is brought down. Furthermore, if the ante-uterine peritoneal space be not opened, the work of securing the lateral vesicular supply must be greatly embarrassed, and the danger of wounding the ureters greatly, almost infinitely, increased.

The second step in the operation consists in hemostasis and it includes securing and dividing the broad ligaments.

The devices that have been used to secure hemostasis are almost legion. Until very recently silk ligatures only were used

---

[a] Deutsche Med. Wochenschrift. Berlin. 1886. xii, 602-604.

to secure the whole mass of the ligaments or to secure it in separate divisions by the continuous or by the loop method. Later the ecraséur, wire or elastic ligature has been used. The cautery has been used. A separate catgut ligature for each tube has been recommended. Needles with a great variety of curves have been devised. The application of ligatures is attended with much difficulty, often failing in the most skilled hands.

By far the best method of accomplishing hemostasis is the snap forceps. It is a sure method; it abbreviates the operation and fords additionally perfect drainage. Before using them it is always well to test the ratchet and ascertain whether they will hold permanently. Occasionally forceps will unsnap, and a greater calamity cannot befall an operation than to have that occur after leaving the patient. Tieing the forceps together when in doubt about their reliability can be done.

After the peritoneal cavity before and behind the uterus has been opened and the uterus has been completely flexed, when possible, and is retained by the *ligamenta lata* only, the latter are ready to receive the forcipressure. With the forefinger of the left hand hooked over the superior margin of the left broad ligament, the right hand can adjust the forceps to compress the whole width of the ligament and tighten the instrument to the last notch. It is best to attach it as near to the uterus as possible, and yet permit room for dividing the ligaments easily at its uterine end. While adjusting the forceps, it is of course scarcely necessary to mention the desirability of not including in them a bit of omentum or a piece of intestine. I know of no greater satisfaction in gynecological operations that the operator can experience than in tightening hemastic forceps on a broad ligament—a satisfaction greatly intensified when one has previously had the appalling accident occur of the shedding of the silk ligatures after the broad ligament has been permitted to contract and withdraw into the pelvis up out of sight.

When the uterus cannot be flexed, the forceps have to be applied in the best way that can be devised. With a much enlarged uterus the forceps can be applied to include broad ligament to the extent of the width of its jaws; that amount of ligament can be divided, and up through the divided segment another pair of forceps can be pushed to include the remainder of the ligament, which in turn can be divided. When the finger cannot reach the superior margin of the ligament, the lower section of each ligament can be seized and divided, when it will be found that the whole organ can be made to descend, and thus the entire ligament upon each side can be divided. When this procedure is necessary the difficulty of practicing is greatly increased because of the narrowing of the vaginal space.

After removing the uterus the parts should be allowed to retract in order to allow any vessels to bleed that are prevented from it by their traction. By this means arterial twigs are often discovered which otherwise escape detection. All further arresting of hemorrhages can be acccomplished easily with forceps. This step in the operation is of vast importance, since hemorrhage cannot only result fatally, but even when not large it can become the unsuspected cause of a fatal peritonitis.

The last step of the operation concerns the management of the womb. The most elaborate sewing and draining of the vaginal cavity has been resorted to. Stitching the peritoneum to the vaginal wall is regarded necesssry by some operators. One operator recently stated, in his report of a case, that he stitched these two tissues together in front of the uterus before opening the Douglas pouch. Stitching the anterior marginal border of the rent to the posterior border, drawing the ends of the ligatures out through their center, has been very commonly done. Running a purse-string suture around the top of the vagina with a piece of rubber draining tube and the ligatures passing through the middle of the puckering has been used.

Sewing up the vagina is wholly unnecessary in most cases. These various closings of the vagina have been regardad as essential to keep back the bowels and to prevent a septicæmia through the vagina. Of the former there is a minimum danger. When the operation is completed the superior vaginal opening collapses as thoroughly and completely as the *ostium vagina* closes. The oozing opposed surfaces at once interdigitate and inaugurate the preliminary processes of union. They do not lie idle for 24

or 48 hours before commencing union is set up. At the end of 48 hours the top of the vagina is all closed to the passage of fluids, excepting through that portion of it occupied by the means of drainage.

The use of iodoform gauze in the vagina is of the utmost importance, and when wrongly used is a source of danger. The vagina must be absolutely aseptic, and herein the gauze filled with iodoform becomes of such great service. Stuffing the vagina too full of this agent keeps apart the walls of the top of the vagina and prevents their union.

---

OBLITERATED SCALE OF CLINICAL THERMOMETERS.—Many students and practitioners are inconvenienced by their clinical thermometers becoming obliterated. I found a good way to renovate them in the American copying pencils so much used by students. To use them, the scale is to be first moistened, then the pencil is to be well rubbed in. When it has become dry, the superfluous coloring matter may be removed by a soft cloth, when the roughened markings of the scale will be found to retain the color which will last just as long as the original marking. I hope some of your readers may find this useful.—*Physician and Surgeon.*

